# Effectiveness of Biomarker-Guided Implant Risk (BGIR) vs. IDRA and PiRA Scores in Predicting the Onset of Peri-Implant Diseases: A Long-Term Retrospective Study

**DOI:** 10.3390/jcm15208159

**Published:** 2026-10-20

**Authors:** Renzo Guarnieri, Rodolfo Reda, Gabriele Miccoli, Marta Cianfriglia, Gualtiero Gherardi, Luca Testarelli, Dario Di Nardo

**Affiliations:** 1 Private Peridontal Implant Practice, 31100 Treviso, Italy; 2 Dental School, Campus Bio-Medico University of Rome, 00128 Rome, Italy; r.reda@unicampus.it (R.R.); g.miccoli@policlinicocampus.it (G.M.); m.cianfriglia@policlinicocampus.it (M.C.); g.gherardi@policlinicocampus.it (G.G.); 3 Department of Oral and Maxillofacial Sciences, Sapienza University of Rome, 00161 Rome, Italy; dario.dinardo@uniroma1.it

**Keywords:** prognosis, peri-implantitis risks, aMMP-8

## Abstract

**Objectives**: The long-term prognostic value of the biomarker-guided implant risk (BGIR) score was compared with two other risk models, the IDRA (implant disease risk assessment) and the PiRA (peri-implantitis risk assessment), in predicting the onset of implant diseases in patients attending a private practice and enrolled in a supportive care program (SCP). **Methods**: From a pool of private implant patients subjected to at least 10 years of SCP, 80 partially edentulous subjects were recruited and retrospectively evaluated. The BGIR, the IDRA and the PiRA scores were calculated during SCP and compared to each other. Prognostic scores of BGIR, IDRA and PiRA were estimated based on the area under the curve for receiver operating characteristic (ROC), including 95% confidence intervals. High-risk BGIR, IDRA and PiRA subgroups versus low/moderate BGIR, IDRA and PiRA subgroups were compared at the patient level using Cox regression analyses. **Results**: The mean implant follow-up was 10.5 years. Peri-implantitis incidence was 17.5%. The BGIR, IDRA and PiRA high-risk percentages were 27.5%,21.2% and 25%, respectively. The areas under the ROC curve for BGIR, IDRA and PiRA were 0.836, 0.758, and 0.769, respectively, for peri-implantitis events. BGIR, IDRA and PiRA high-risk scores were found to be significantly associated with the onset of peri-implantitis, with hazard ratios (HR) of 4.7, 3.6 and 3.8, respectively. Compared to IDRA and PiRA, BGIR showed higher specificity and sensitivity. However, comparisons between the area under the, curves (AUC) of the three independent ROC curves showed no statistical significance. **Conclusions**: Compared to IDRA and PiRA, calculation of the BGIR score during follow-up seems to have a greater ability to predict the onset of peri-implant diseases in daily clinical practice.

## 1. Introduction

**Figure 1:**
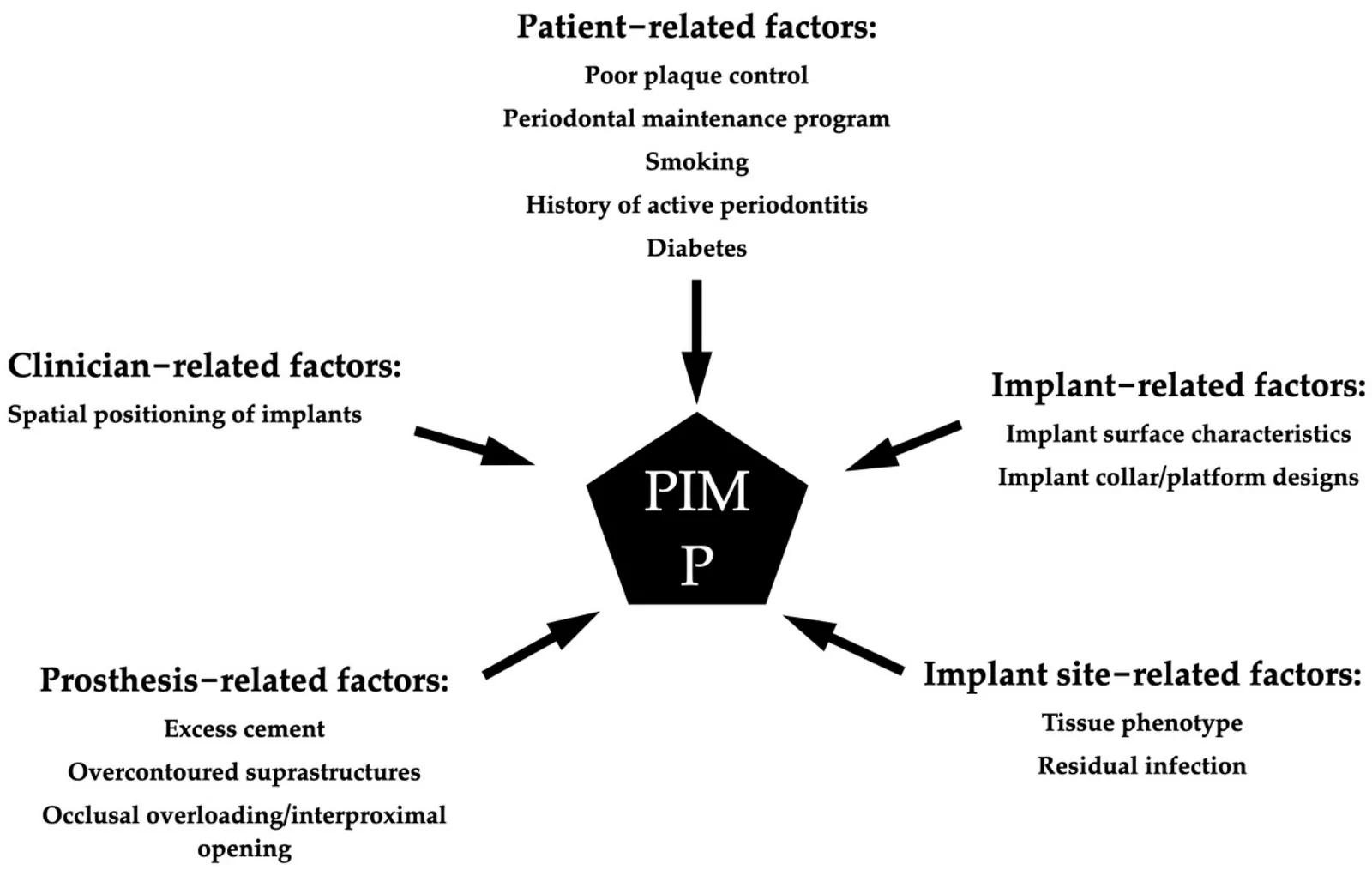
Summary of factors to control for in avoiding peri-implant inflammatory diseases. Modified from Fu and Wang [6].

Prevention of peri-implant inflammatory diseases, such as peri-implant mucositis (PIM) and peri-implantitis (P), is considered one of the most important issues in modern dental implantology [1,2]. Recent analyses estimated that the mean prevalence of PIM and PI is approximately 43% and 22% in Europe and South and North America, and 19% and 11.2% in China, respectively [3,4]. The current literature identified several factors that could increase or reduce the onset of these diseases [5,6], which can be broadly classified into five groups: (1) patient, (2) implant design, (3) implant site, (4) prosthesis, and (5) clinician-related factors (Figure 1). These factors interact in a complex way, exerting a synergistic action on the host’s overall response to bacterial plaque at implant sites [5,6]. The implant disease risk assessment (IDRA) [7] and peri-implantitis risk assessment (PiRA) [8], which combine some of the various parameters identified, have been previously proposed to predict the risk of developing peri-implant diseases.

Several retrospective studies testing these tools (particularly the IDRA) have suggested that they have good accuracy in determining the risk of developing P [9,10,11]. However, the relative heterogeneity of the parameters included in the calculation of IDRA and PiRA risk scores and the subjective choice (mostly dependent on expert opinions) raise a question regarding the selection criteria for the parameters and calculation methods, as already discussed for periodontal diseases [12]. Furthermore, the use of the above-mentioned tests is time-consuming and does not seem practical when the intention is to perform a periodic re-evaluation of the conditions of the peri-implant tissues.

For the early detection of inflammation in peri-implant tissues and for the potential predictive assessment of the onset of peri-implant diseases, in recent years, there has been growing attention to biomarkers present in peri-implant sulcular fluid (PISF) [13,14,15,16,17,18]. One of the most studied biomarkers is active metalloproteinase-8 (aMMP-8), whose most important physiological role is the degradation of various collagen types (types I and III), extracellular matrix proteins, complement factors, and other biomolecules [19,20]. Many studies investigating the correlation between the aMMP-8 concentration in PISF and clinical indices of peri-implant inflammation, such as bleeding on probing (BoP), probing depth (PD), clinical attachment loss (CAL), and radiographic marginal bone loss (MBL), have demonstrated its sensitivity, specificity, and diagnostic accuracy [21,22,23]. Therefore, measurement of the PISF aMMP-8 concentration has been proposed as an indicator capable of identifying, early and often, invisible alterations in implant-supporting structures before clinical and radiological signs document a full-blown lesion. A point-of-care (POC)/chairside test (ImplantSafe, Dentognostics GmbH, Solingen, Germany) has recently become available, allowing the identification and quantification of the presence of aMMP-8 in the peri-implant sulcular fluid (PISF) quickly and easily [23,24]. Since for effective prevention of peri-implant inflammatory diseases, a simplified, biologically driven framework is needed to support clinicians in defining individualized maintenance plans, a biomarker-guided implant risk (BGIR) tool has been recently proposed [25,26]. In addition to patient-, implant-, and prosthesis-related risk factors/indicators, including the intrasulcular aMMP-8 dosing, it is designed to integrate a rapid chairside procedure into the evaluation.

In a previous study, the authors evaluated—in the same patients included in the present study—both the potential efficacy of the BGIR tool in predicting the onset of peri-implantitis (P) and the hazard ratios (HR) for each individual parameter considered in its determination [26]. The individual parameters hazard ratio (HR) comparison for P events showed a higher value (HR = 6.21, *p* = 0.003) for aMMP-8 > 80 ng/mL, allowed by history of stage II, III and IV periodontitis (HR = 5.19 (*p* = 0.006)), while the other parameters individually were not found to be significantly associated with P, thus showing the PISF aMMP-8 concentration to be of better prognostic significance.

Since, compared to the other parameters considered, the intra-sulcular concentration of aMMP-8 was found to have a high HR, the aim of the present study was to compare the effectiveness of the BGIR score with respect to other indices (IDRA and PiRA), which only involve the use of clinical and radiological indices in their determination.

## 2. Materials and Methods

This retrospective study was approved by the Ethics Committee for Health (prot. no. 4597) of Sapienza University of Rome and follows the Helsinki Declaration of 1975, as revised in 2000. The patients provided their informed consent to participate in the study. The study was conducted at a private rehabilitation center (Treviso, Italy) concerning patient rehabilitation and follow-up between January 2005 and December 2015 (data collection, analysis and interpretation).

### 2.1. Studied Population

Inclusion criteria were

(a) patients rehabilitated with at least one dental implant, followed for at least 10 years, and irrespective of their systemic health condition and smoking status;
(b) age > 18 years;
(c) the presence of a stable periodontal condition;
(d) completeness of clinical data collected during follow-up.

Exclusion criteria were

(a) absence of medical records with complete data and follow-up for at least 10 years;
(b) patients who were deceased;
(c) patients in need of antibiotic prophylaxis for oral examination and treatments.

Sample Size: The final sample included a total of 80 patients (51 female, 29 male) with an average age of 55.1 years (standard deviation: 12.75 years; age range: 21 to 71 years) and 138 implants (BioHorizons Tapered Internal Laser-Lok system, Birmingham, AL, USA).

### 2.2. Follow-Up

During follow-up, each patient underwent a check-up at intervals of 3 to 6 months, depending on periodontal and implant conditions, and a complete clinical evaluation was performed annually. Two years of lack of follow-up visits during SCP were considered to classify patients as non-compliant.

Periodontal and implant examinations were performed by experienced periodontists (RG, LT). Full-mouth periodontal pocket depth (PPD), bleeding on probing (BoP), and suppuration were recorded. All the measurements were performed at six points of each tooth and implant using a PCPUNC 15 probe (HuFriedy, Chicago, IL, USA).

According to the guidelines proposed by the American Academy of Periodontology/European Federation of Periodontology 2017 World Workshop on the Classification of Periodontal and Peri-implant Diseases and Conditions [27], the diagnosis of P was made on the basis of progressive peri-implant bone resorption, beyond the initial physiological remodeling, on the presence of an increase in probing depth accompanied by bleeding and/or suppuration.

PISF sample analysis: after removing excess saliva with a gentle blast of air around the site, the PISF was collected using a strip of sterile absorbent paper placed as deeply as possible into the peri-implant sulcus. The PoC/chairside mouth rinse test (Implant/Safe^®^, Biomolecular Diagnostic Sr, Firenze, Italy) and a digital reader (ORALyzer^®^ Dentodiagnostic GmbH, 42699 Solingen, Germany) were used, according to the manufacturers’ indications, to determine the doses of aMMP-8.

Determination of PISF aMMP-8 levels and BGIR, IDRA and PiRA risk score calculations were performed during the 3-year follow-up visit. Events of P were counted if they were diagnosed during the entire follow-up period.

### 2.3. Radiographic Examination

Radiological examinations were performed using digital radiographs obtained with a 70 kW/7.5 mA X-ray machine. Linear changes in the MBL were calculated using dedicated software (DB-SWIN 2.0 software, Durr Dental Italy S.r.l, Muggiò, Italy), taking as reference the measurements recorded at implant placement, at prosthesis delivery (6 months), and at each annual visit. At the same time points, the number of sites with plaque (P), probing depth (PD), the number of sites with bleeding on probing (BoP), the full-mouth plaque score (FMPS), and the full-mouth bleeding score (FMBS) were measured.

**Assignment of BGIR score**

(1) aMMP-8 level. A POC/chairside test is used to measure the concentration of aMMP-8 in the PISF. Based on what was proposed by Lähteenmäki et al. [27], three risk categories have been proposed based on the levels of aMMP-8 recorded:
  (a) low risk (aMMP-8 level < 20 ng/mL),
  (b) moderate risk (aMMP-8 level between 20 and 80 ng/mL),
  (c) high risk (aMMP-8 level > 80 ng/mL) [28].
(2) Oral hygiene status:
  (a) low-risk plaque score = presence of ≤l site around implant with a plaque index (PI) > 1 and absence of poor fit and/or cement excess [29],
  (b) moderate-risk plaque score = presence of 1 or 2 sites with PI > 1 with no poor fit and/or cement excess,
  (c) high-risk plaque score = presence of 3 or more sites with PI > 1 and/or with poor fit and/or cement excess.

The radiographic presence of an open margin between the abutment and restoration was used to identify a poor fitting condition [30].

(3) History of periodontitis: According to the 2017 World Workshop on the Classification of Periodontal and Peri-Implant Diseases and Conditions [31], history of periodontitis was considered in the presence of pre-treatment diagnoses of stage II, III, and IV periodontitis [30]. Patients with an initial diagnosis of stage II, III, or IV periodontitis were classified into moderate-risk and high-risk categories. Patients with an initial diagnosis of gingivitis or stage I periodontitis were considered not to have a history of periodontitis and classified into the low-risk category.
(4) Smoking:
  (a) low-risk score is assigned in cases with no cigarette consumption;
  (b) moderate-risk score is assigned in cases with previous cigarette consumption;
  (c) high-risk score is assigned in cases with cigarette consumption [32].
(5) Systemic diseases: low-risk score is assigned in cases not having systemic diseases; moderate-risk score as assigned in cases not having or having systemic diseases with good control; high-risk score is assigned in cases having systemic diseases [33].
(6) Prosthetic design related to homecare hygiene procedure: low-risk score is assigned in presence of prosthetic emergence angles (EA) </= 30° and a concave emergence profile; moderate-risk score is assigned in presence of EA > 30° (regardless of concave or convex profile), or EA </= 30°associated with convex profile; high-risk score is assigned in presence of EA > 30° associated with convex profile [34,35].
(7) Patient dexterity assessment: low-, moderate- and high-risk scores are assigned in presence of good, limited and compromised patient dexterity, respectively.
(8) Supportive care program (SCP) compliance: only two levels of compliance were used to determine patient adherence to SCP before score calculation [36,37,38].
  (a) presence of non-compliance, defined as no maintenance visit for at least 2 years = moderate-/high-risk score
  (b) presence of good compliance with SCT = low-risk score.

**Figure 2:**
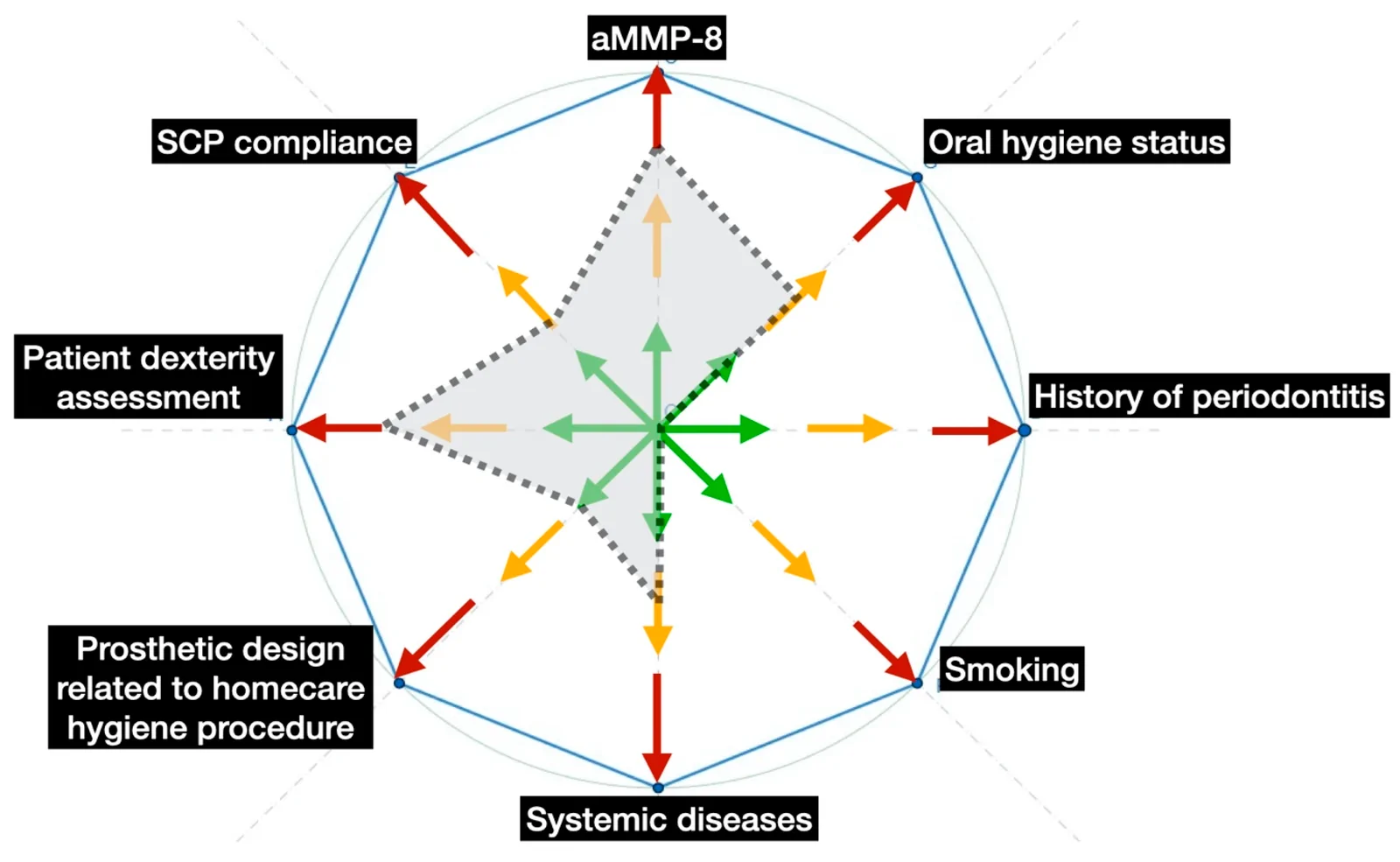
Example of a BGIR risk calculation: Green = low risk, yellow = moderate risk, red = high risk. High BGIR patients = those with aMMP-8 level > 80 ng/mL and limited patient dexterity.

For BGIR risk assessment scores, low-risk patients have aMMP-8 level < 20 ng/mL and at least six parameters in the low-risk categories or only one in the moderate-risk category. A patient with a moderate BGIR score could also have an aMMP-8 level between 20 and 80 ng/mL, and at least 6 parameters in the moderate-risk category or one parameter in the high-risk category. Patients with a high BGIR score have an aMMP-8 level > 80 ng/mL and at least one parameter in the high-risk category or six parameters in the moderate-risk category. Figure 2 reports an example of the BGIR score calculation.

**Assignment of IDRA risk [7].**

The categories and parameters as described by the authors [7] for the determination of the IDRA risk are reported below.

(1) History of periodontitis: It is assessed anamnestically and/or by evaluating radiographic bone loss around the teeth [7].
(2) Percentage of sites with bleeding on probing (BoP): It is evaluated for all teeth or implant sites present. In the presence of a percentage of BoP < 10%, the patient is classified as low risk, while in the presence of BoP > 25%, the patient is considered at high risk [7,39].
(3) Prevalence of PD ≥ 5 mm: In the presence of 1 or 2 pockets with PD ≥ 5 mm, the patient is considered at low risk, while if there are more than 6 pockets with PD ≥ 5, the patient is considered at high risk [7].
(4) Periodontal bone loss in relation to the patient’s age (BL/age): Periodontal bone loss is estimated radiographically at the most affected site as a percentage of root length, calculating that 1 mm of bone loss roughly corresponds to 10%. To obtain the BL/age ratio, the estimated percentage is divided by the patient’s age. If the BL/age ratio is >1, the patient is considered at high risk [7].
(5) Periodontitis susceptibility: Patients with an initial diagnosis of stage II, III, and IV periodontitis [31] were classified into the moderate, moderate/high and high-risk category, respectively. Patients with an initial diagnosis of gingivitis or stage I periodontitis were considered not to have a history of periodontitis and classified into the low-risk category [7].
(6) Supportive periodontal therapy (SPT): If the patient adheres to an SPT program, he or she is considered at low risk, while patients non-adherent to SPT are considered at high risk [7].
(7) Distance from the restorative margin (RM) of the implant-supported prosthesis to the bone: It is assessed in millimeters on the X-ray taken at the time of delivery of the prosthesis or, if not available, on the X-ray taken when the risk score is determined. Tissue-level implants are considered at low risk. Implants with an RM > 1.5 mm are considered at moderate risk, and those with an RM < 1.5 mm are considered at high risk [7].
(8) Implant prosthesis-related factors: A well-tightened, easily cleanable prosthesis free of excess submucosal cement is considered at low risk.

A poorly tightened but easily cleanable prosthesis free of excess submucosal cement is considered at moderate risk. A poorly tightened prosthesis with excess submucosal cement and/or poorly cleanable is considered at high risk [7].

**Figure 3:**
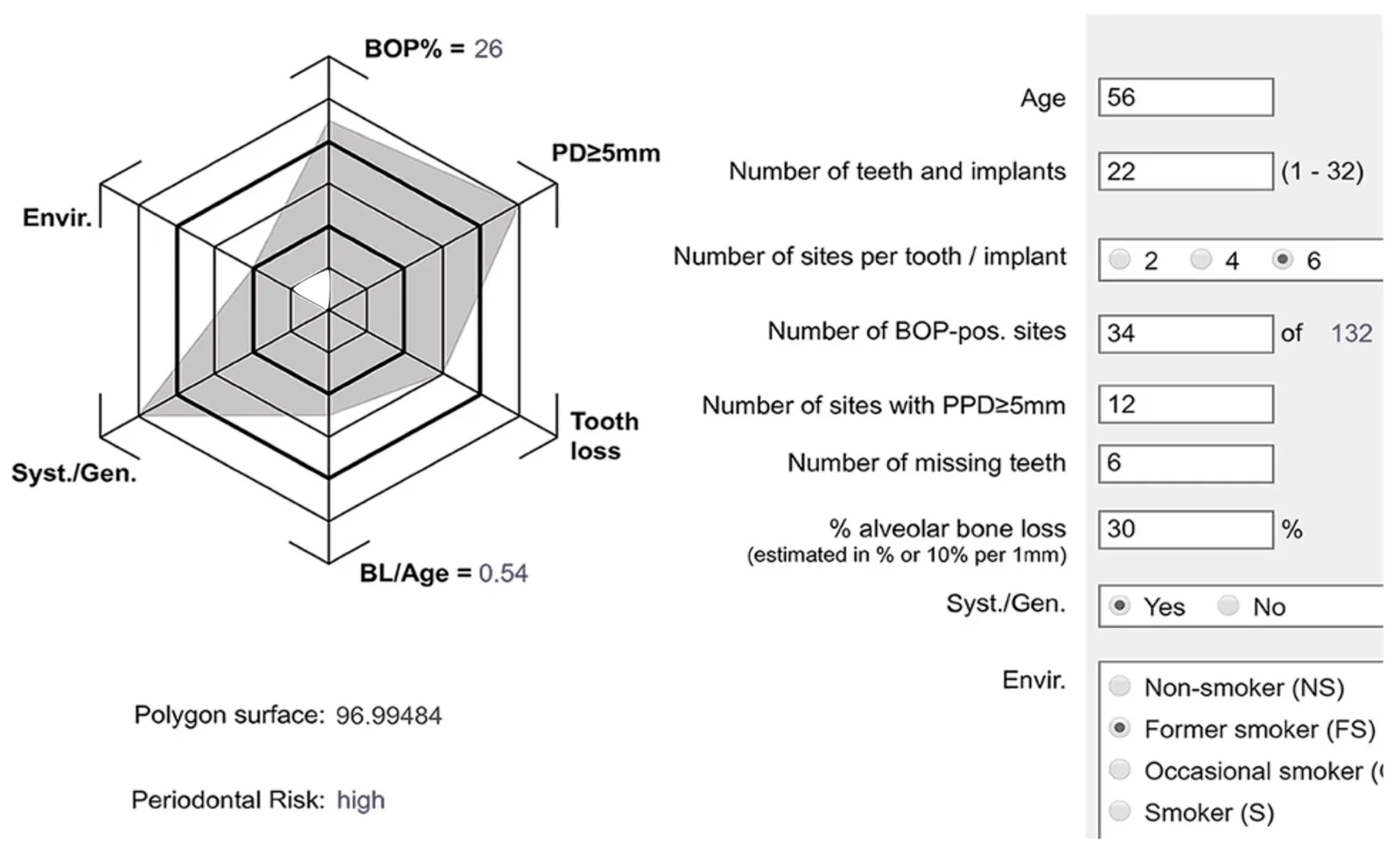
Example of a IDRA risk calculation. Modified from Heitz-Mayfield et al. [7].

IDRA score determination: If all parameters are rated at low risk or only one parameter is rated at moderate risk, the patient is considered at low risk. If only one parameter is rated at high risk, even if all other parameters are rated at low risk, the patient is considered at moderate risk. If at least two parameters are rated at high risk, the patient is considered high risk [7]. An online tool implementing the model is available: https://www.perio-tools.com/idra/?lang=it (accessed on 12 July 2020). Figure 3 reports an example of the IDRA score calculation.

**Assignment of PiRA risk [8]**

The categories and parameters as described by the authors for the determination of PiRA risk are reported “textually” below.

(1) Periodontitis susceptibility: It is determined from the bone loss/age ratio. A patient who has lost a higher percentage of the alveolar bone relative to his/her age is at high risk [8].
(2) Smoking: A low-risk score is assigned in cases with no cigarette consumption; a moderate-risk score is assigned for cigarette consumption between 0 and 19, while a high-risk score is assigned for cigarette consumption > 20 [8].
(3) Full mouth bleeding score: A low-risk score is assigned in cases where the percentage of sites displaying BoP < 9; a moderate-risk score is assigned in cases where the percentage of sites displaying BoP > 9 ≤ 19; while a high-risk score is assigned in cases where the percentage of sites displaying BoP > 20 [8].
(4) Number of teeth lost (excluding third molars): A low-risk score is assigned in cases where the number of teeth lost ≤ 4; a moderate-risk score is assigned in cases where the number of teeth lost > 4 ≤ 8; while a high-risk score is assigned in cases where the number of teeth lost > 8 [8].
(5) Number of sites with probing pocket depth (PPD) ≥ 5 mm: A low-risk score is assigned in cases where the number of sites with PPD ≥ 5 mm ≤ 4; a moderate-risk score is assigned in cases where the number of sites with PPD ≥ 5 mm > 4 ≤ 8; while a high-risk score is assigned in cases where the number of sites with PPD ≥ 5 mm > 8 [8].
(6) Recall frequency: It is determined by the number of months before recalls. A low-risk score is assigned in cases where the number of months before recalls ≥ 6; a moderate-risk score is assigned in cases where the number of months before recalls > 6 ≤ 8; while a high-risk score is assigned in cases where the number of months before recalls > 12 [8].
(7) General health (diabetes): It is determined by only two levels: yes/no [8].

An online tool implementing the model is available: https://pira.gbiomed.kuleuven.be/ (accessed on 12 July 2020).

**Figure 4:**
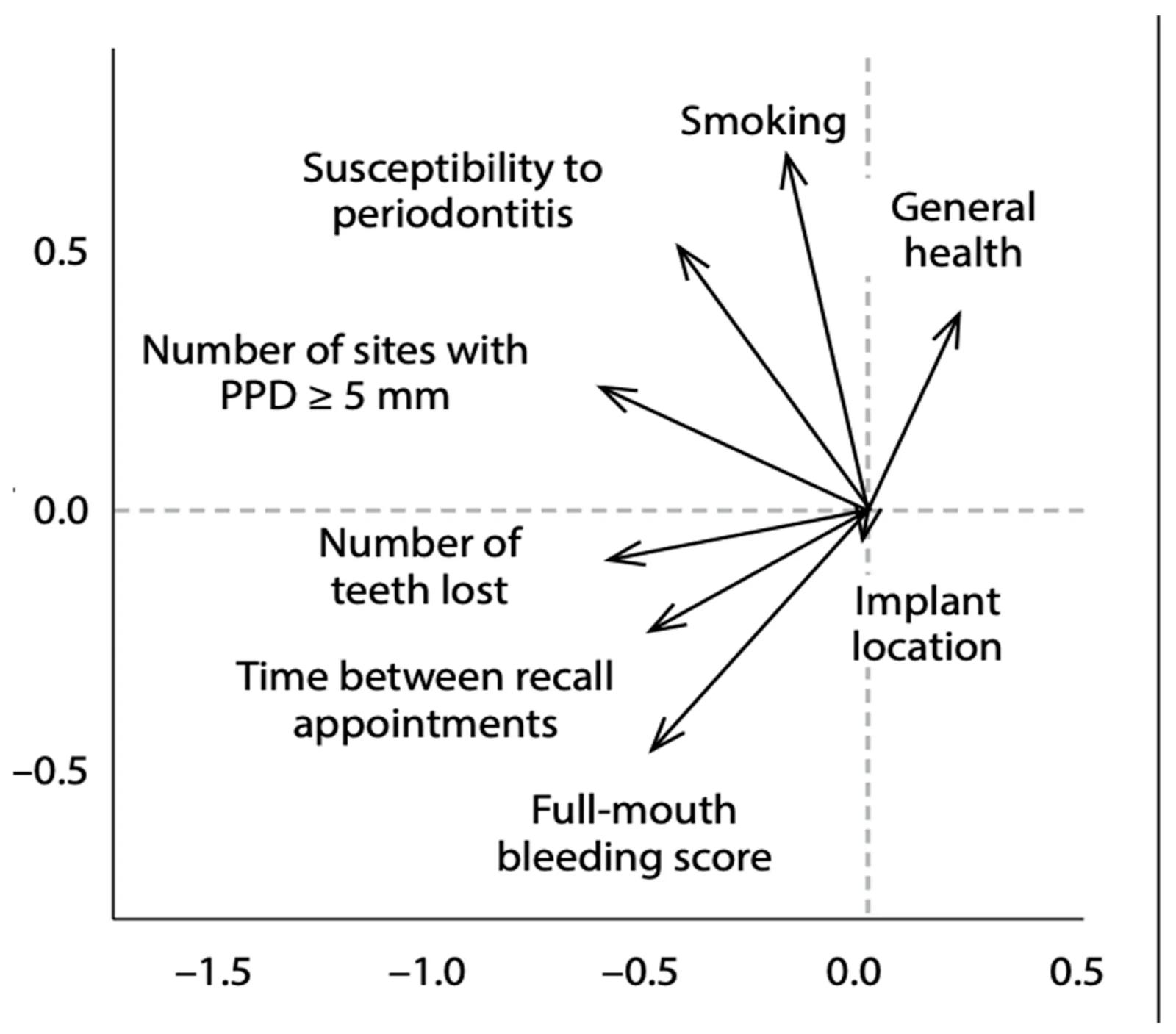
Example of PiRA calculation. Modified from Quirynen et al. [8].

Figure 4 reports an example of the PiRA score calculation.

### 2.4. Statistical Analysis

Statistical analysis was performed at patient level. Diagnosis of P during follow-up was defined as the primary endpoint for analysis. The discriminatory ability of BGIR, IDRA, and PiRA was evaluated using receiver operating characteristic (ROC) curves and the related determinations of sensitivity, specificity, and positive and negative predictive values. Sensitivity was derived from positive predictive values, negative predictive values, and likelihood ratios. Likelihood ratios describe the odds of developing P in a patient for which the model predicts P. Univariate and multivariate Cox regression analyses estimating hazard ratios (HR) with 95% confidence intervals were performed for each evaluated parameter and for risk score. Occurrence time of P was defined as the first occurrence of P per patient after BGIR, IDRA and PiRA calculation. Ridge regression (*L*_2_ penalty) and Lasso regression (*L*_1_ penalty) were used to shrink unstable coefficients toward zero and to prevent overfitting with multiple parameters. Agreement between high risk measured by BGIR, IDRA, and PiRA was assessed using Cohen’s kappa coefficients. A *p* < 0.05 difference was considered as significant.

The comparison of three ROC curves and the related AUC (area under the curve) values was performed using the Hanley–McNeil method.

All statistical calculations were performed and figures created using Statistic Kingdome Calculators (https://www.statskingdom.com) (accessed on 21 April 2026).

## 3. Results

The mean age was 60.09 years at score calculation time. The numbers of no smokers, past smokers, occasional smokers and smokers were 39 (48.7%), 16 (20%), 4 (5%), and 21 (26.3%), respectively.

Seven patients (8.7%) were diagnosed with uncontrolled systemic diseases (2 diabetes, 5 hypertension). Eleven patients (13.7%) presented controlled systemic diseases (1 diabetes, 4 hypertension, 3 hyperthyroidism, 2 hypothyroidism, 1 hypercholesterolemia). A total of 60 patients (75%) had a history of periodontitis: 13 (16%) Stage II—Grade A/B, 34 (42.5%) Stage III—Grade A/B, 6 (7.5%) Stage III—Grade C, and 7 (8.8%) Stage IV. Sixty-two patients (77.5%) were considered compliant, and 18 (22.5%) non-compliant. Fifty-eight patients (72.5%) presented good dexterity, 12 (15%) limited dexterity, and 10 (12,5%) compromised dexterity. Fifty-three (66.2%), 24 (30.1%), and 3 (3.7%) patients presented low-risk, moderate-risk, and high-risk plaque scores, respectively. No patients had excess cement, and only two patients (2.5%) presented an implant with supra-mucosal poor fit. Forty-six patients (57.5%) presented EA </= 30° and concave emerging profile with easy access, 21 (26.2%) presented EA > 30° with limited access, and 13 (16.3%) presented EA > 30° and convex profile, associated with difficult access. PISF aMMP-8 values were <20 ng/mL, between 20 to 80 ng/mL, and >80 ng/mL in 58 (62.5%), 14 (27.5%), and 8 (10%) patients, respectively.

Recorded tooth loss was 8.44%, BoP was 26.25% (present in 32.88% of patients), and the number of sites with PD > 5 mm was 26.4%. Twenty-five patients (31.2%) presented PD > 5 mm in more than eight sites, and 26 patients (32.5%) presented PD > 5 mm in more than six sites. BL/age > 1 was found in 16.25% of patients, and the mean BL/age was 0.79.

No patients had P before score determination, and at the end of follow-up, P was diagnosed in 14 patients (17.5%).

**Figure 5:**
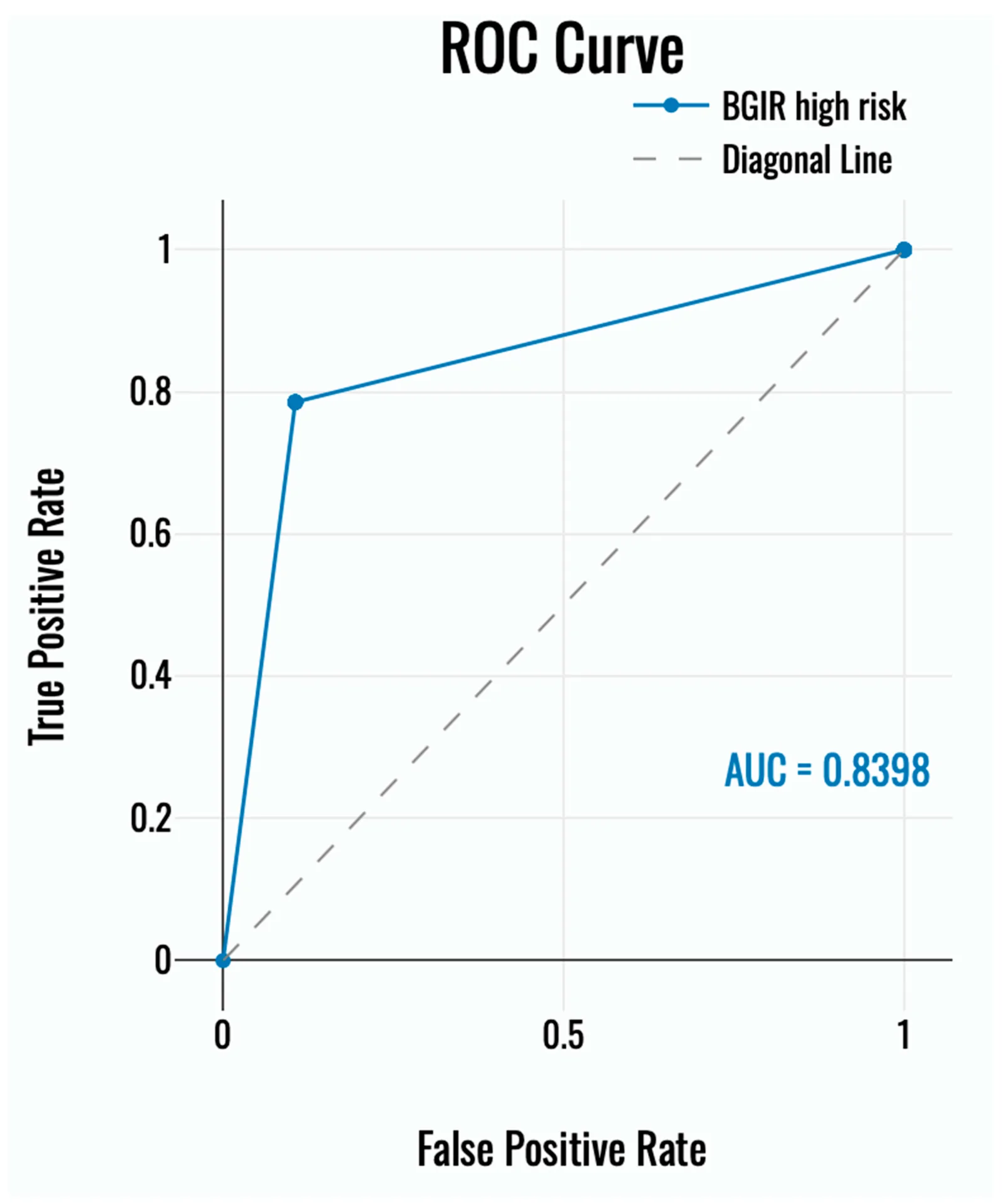
ROC curve illustrating the performance of BGIR.

**BGIR risk score**: Seven patients (8.7%) presented a low-risk BGIR score, while moderate-risk BGIR patients represented 51 (63.7%) cases, and high-risk BGIR patients represented 22 (27.6%) cases. Concerning agreement between scores for risk evaluation, high-risk BGIR matched in 22 patients (Cohen’s kappa coefficient 0.611). Table 1 reports patient data at BIGR calculation time. The area under the ROC curve (AUC) was 0.836, 95% CI [0.72, 0.95], SE = 0.06, *p* < 0.001 for P events (Figure 5). The sensitivity, specificity, accuracy, positive predictive value, and negative predictive value were 0.7857 CI [0.52, 0.92], 0.8939 [0.8, 0.95], 0.875 [0.79, 0.93], 0.6111 [0.39, 0.8], and 0.9516 [0.87, 0.98], respectively.

Multivariable Cox regression analyses showed that high-risk BGIR patients were 4.7 times (HR = 4.7) more likely to have P during follow-up. Figure 5 shows the area under the ROC curve (AUC) in relation to the individual variables considered.

**Figure 6:**
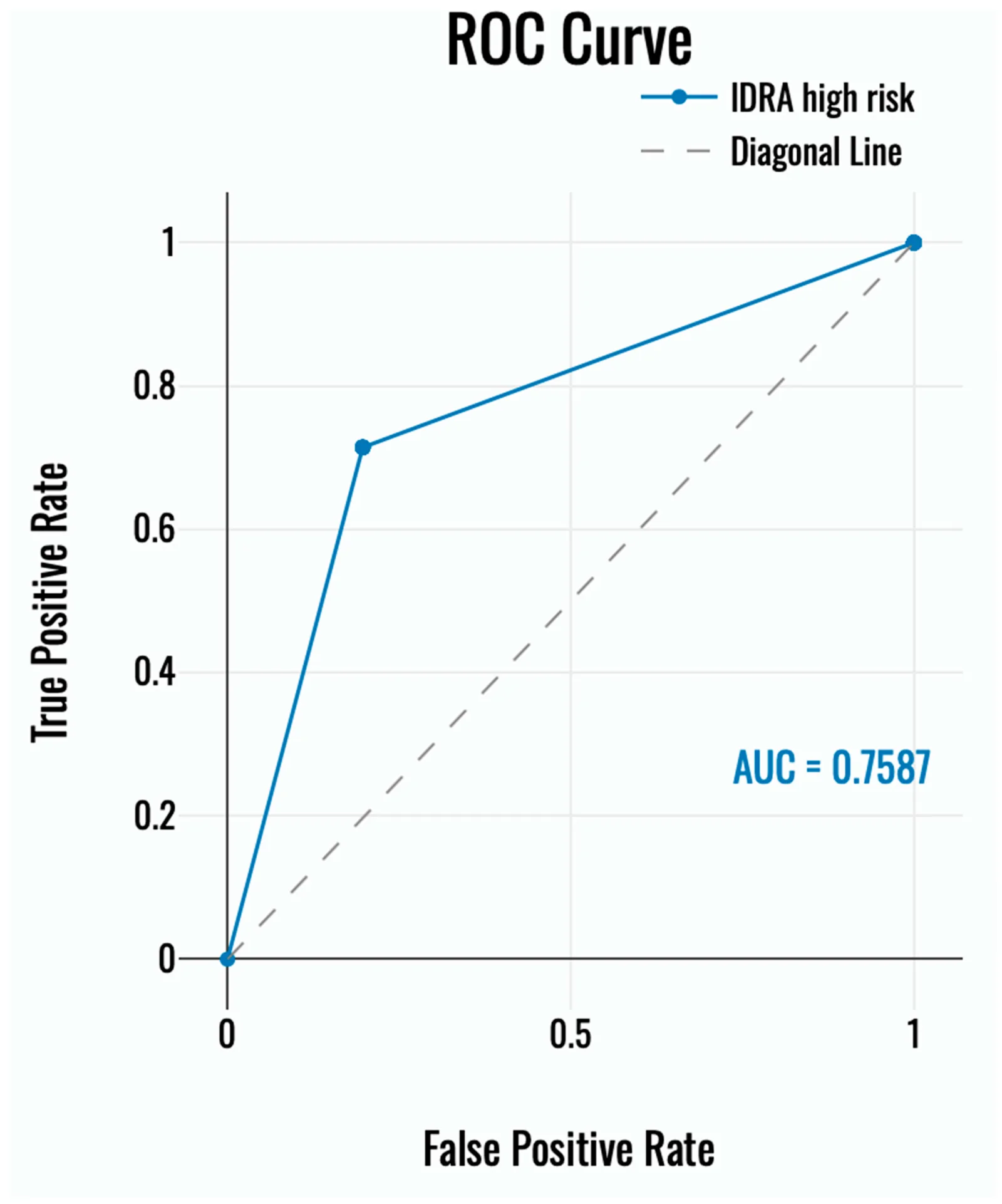
ROC curve illustrating the performance of IDRA.

**IDRA risk score:** Fifteen (15) patients (18.7%) presented a low-risk IDRA score, while 46 (57.5%) presented a moderate-risk IDRA score, and 17 (21.2%) a high-risk IDRA score. Concerning agreement between scores for risk evaluation, high-risk IDRA matched in 17 patients (Cohen’s kappa coefficient 0.598). Table 2 reports patient data at IDRA calculation time. The area under the ROC curve (AUC) for IDRA was 0.758, 95% CI [0.63, 0.89], SE = 0.07, *p* < 0.001 for P events. The sensitivity, specificity, accuracy, positive predictive value, and negative predictive value were 0.557, CI [0.41, 0.66], 0.721, CI [0.6, 0.81], 0.525 [0.42, 0.59], 0.611 [0.39, 0.8], and 0.551 [0.47, 0.63], respectively. Multivariable Cox regression analyses showed that high-risk BGIR patients were 3.6 times (HR = 3.6) more likely to have P during follow-up. The sensitivity, specificity accuracy, positive predictive value, and negative predictive value were 0.557, CI [0.41, 0.66], 0.721, CI [0.6, 0.81], 0.525 [0.42, 0.59], 0.611 [0.39, 0.8], and 0.551 [0.47, 0.63], respectively. Multivariable Cox regression analyses showed that high-risk BGIR patients were 3.6 times (HR = 3.6) more likely to have P during follow-up. Figure 6 shows the area under the ROC curve (AUC) in relation to the individual variables considered. Twenty-five patients (31.2%) presented PD > 5 mm in more than eight sites, and 26 (32.5%) presented PD > 5 mm in more than six sites.

**PiRA risk score**: Eighteen patients (22.5%) presented a low-risk PiRA score, 42 (52.5%) a moderate-risk PiRA score, and 20 (25%) a high-risk PiRA score.

**Figure 7:**
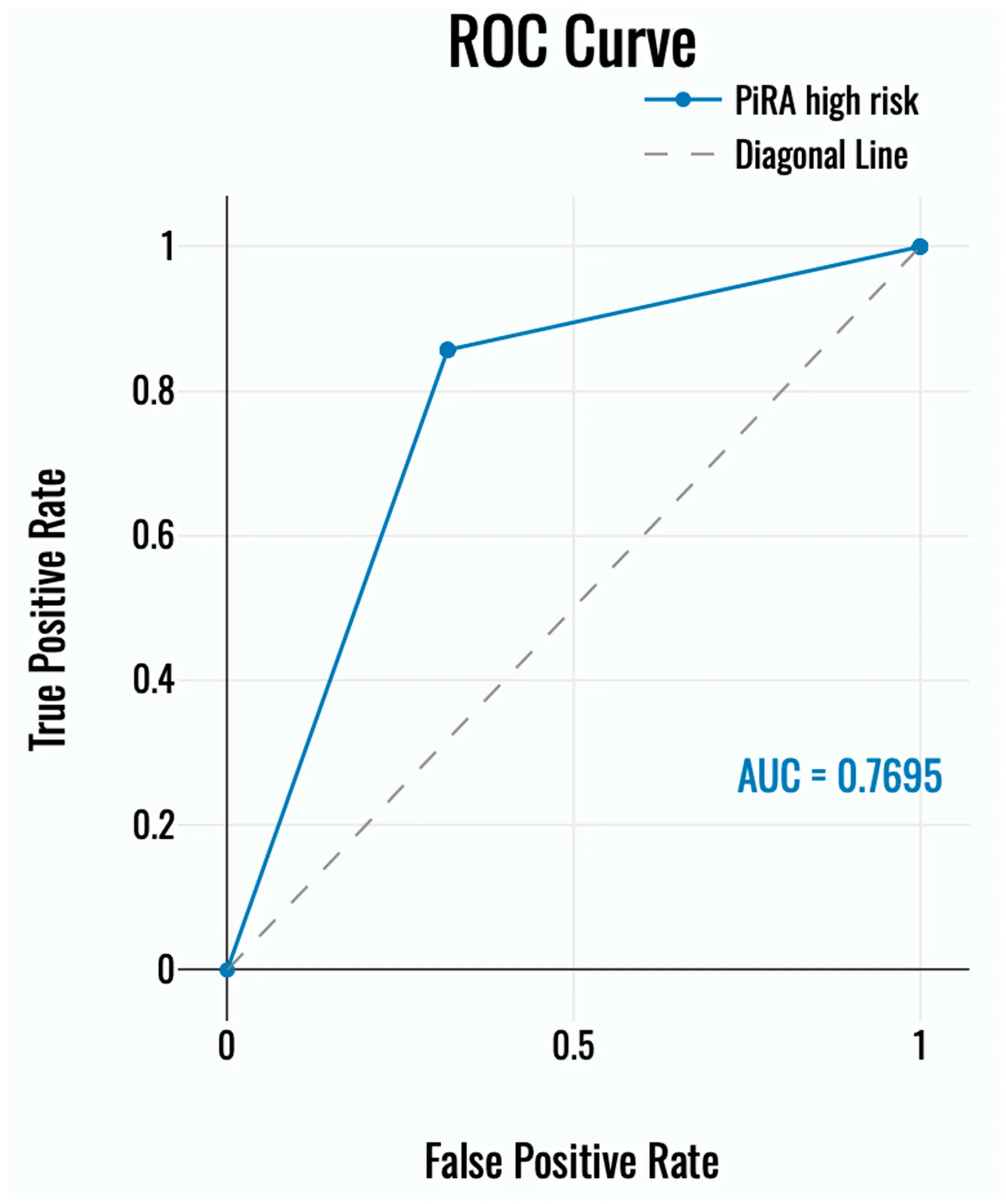
ROC curve illustrating the performance of PiRA.

Concerning agreement between scores for risk evaluation, high-risk PiRA matched in 215 patients (Cohen’s kappa coefficient 0.677). Table 3 reports patient data at PiRA calculation time. The area under the ROC curve (AUC) for PiRA was 0.769, 95% CI [0.66, 0.88], SE = 0.06, *p* < 0.001 for P events. The sensitivity, specificity, accuracy, positive predictive value, and negative predictive value were 0.622, CI [0.48, 0.74], 0.799, CI [0.64, 0.92], 0.582 CI [0.44, 0.70], and 0.881 CI [0.66, 0.91], respectively. Multivariable Cox regression analyses showed that high-risk BGIR patients were 3.8 times (HR = 3.8) more likely to have P during follow-up. Figure 7 shows the area under the ROC curve (AUC) in relation to the individual variables considered.

The comparison of three ROC curves and the related AUC (area under the curve) values using the Hanley–McNeil method did not highlight statistically significant differences in performance between the three models (BGIR vs. IDRA *p* = 0.3790; BGIR vs. PiRA *p* = 0.4458, IDRA vs. PiRA *p* = 0.9131).

## 4. Discussion

The identification of patients potentially at high risk of developing peri-implant inflammatory diseases, after rehabilitation with dental implants, is one of the primary objectives of modern implant dentistry, which in recent years has made significant progress in planning individualized prevention strategies.

To this aim, some research groups have proposed prognostic risk indices, such as the IDRA and PiRA, which provide a quantification of risk factors/indicators for biological complications associated with dental implants. IDRA and PiRA scores combine various factors recognized as predisposing to the onset of P [2,5,6], with associated risk scales, which can be easily carried out online. They have been tested in several retrospective studies that have documented their effectiveness in determining the risk of P [9,10,11]. These risk indices, however, have numerous limitations: the selection of some risk factors and risk thresholds is based on expert opinion, and the risk factors/indicators used have predictive value only when combined, while few associations with the incidence of P have been demonstrated when considered individually [10], and they do not incorporate dynamic biological information derived directly from peri-implant tissues.

In the present retrospective study, a recently proposed risk score (BGIR) was compared with IDRA and PiRA, evaluating its effectiveness in predicting the risk of a patient developing peri-implant disease. Compared with IDRA and PiRA, the major innovation introduced with the BGIR score concerns the intrasulcular dose of aMMP-8, considered a reliable indicator capable of identifying, early and often, invisible alterations in implant-supporting structures before clinical and radiological signs document a full-blown lesion.

Based on collected data, results demonstrate that, compared with IDRA and PiRA, BGIR allowed better identification of patients attending private practice who may develop P in the future. Although comparisons of the area under the curve (AUC) for the three independent ROC curves (BGIR vs. IDRA vs. PiRA) did not show statistical significance, the BGIR score exhibited higher indices of sensitivity, specificity, accuracy, positive predictive value, and negative predictive value compared with IDRA and PiRA.

Some parameters included in IDRA calculation, such as, percentage of sites with BOP, prevalence of PD ≥ 5 mm, and periodontal bone loss in relation to the patient’s age, and in PiRA, such as, full mouth bleeding score and number of teeth lost, are parameters that have demonstrated prognostic value in the periodontal field [40,41,42,43], while they could only indirectly reflect implant risk factors/indicators for P [44,45]. Animal and human studies demonstrated that PD depends on the thickness of the soft tissue at the time of implant placement and on the anatomical circumstances [46]. Moreover, long-term clinical studies have clearly shown that the PD of healthy peri-implant mucosa is not always smaller than 4 mm but very often up to 6 mm [47,48]. For these reasons, there is no specific PD value that can indicate disease conditions, so much so that in the new classification scheme of periodontal and peri-implant diseases and conditions, it is stated that “it is not possible to define a range of PD compatible with peri-implant health” [39]. It is universally accepted that the key parameter for establishing a diagnosis of P is bone loss [39]. Weber et al., evaluating the ability of PD to predict crestal bone loss detected on radiographs in 112 ITI implants, reported no bone changes between years one and five in comparison to increasing PD values during 5 years [48]. Healthy peri-implant soft tissues also have been reported in sites with increased PD values by Giannopoulou et al. in a 9-year follow-up of 61 maxillary anterior implants using clinical, microbiologic and biochemical parameters [49]. Since an increase in PD around implants is not necessarily related to peri-implant bone loss, it is possible to assume that PD does not appear to be a validated diagnostic and prognostic tool.

According to the recommendations of the Consensus report of workgroup 4 of the 2017 World Workshop on the Classification of Periodontal and Peri-Implant Diseases and Conditions [50], BoP is indicated for use as a diagnostic parameter for the identification of both PIM and P. However, recent studies have shown that it is not a reliable indicator of P [45,51]. In fact, it has been documented that the degree of BoP also depends on the type of implant (two-piece/single-piece), the type of abutment material, the position of the implant (anterior/posterior), the sex of the patient and the PD of the probed site, without correlation with the presence of disease [51]. A recent review demonstrated that for BoP-positive implants, there was a 24.1% chance of being diagnosed with P, while for BoP-positive patients, there was a 33.8% probability of being diagnosed with P [52].

The evidence of an association with P remains equivocal also with regard to susceptibility to periodontitis (history of disease, number of teeth lost, periodontal bone loss in relation to the patient’s age) since several systematic reviews on the topic reached no consensus regarding the potential association between periodontitis and P [53,54,55,56,57,58,59,60,61].

Although consensus meetings, which often commission reports, continue to recommend the use of periodontal indices around implants, it is necessary to underline that recent review articles [51,52] and prospective studies [48,49] conclude that periodontal indices are unreliable tools for implant diagnosis and prognosis. IDRA and PiRA scores, which use many periodontal-derived clinical factors as risk assessment tools for peri-implant disease, are also relatively complex and time-consuming, as they have not been specifically designed for repeated chairside use during routine maintenance visits. In daily clinical practice, there is a need for a simplified and biologically driven framework that can support clinicians in determining personalized maintenance programs. The BGIR score was designed to provide clinicians with a simplified, biologically driven framework that can overcome the shortcomings of previously proposed risk scores. In addition to the measurement of some clinical parameters that are considered prognostically important, BGIR involves the measurement of PISF a-MMP-8 concentration by means of POC/chairside test. The choice to use the concentration of aMMP-8 in PISF arises from the need to be able to use an indicator capable of identifying the early and often invisible alterations to implant-supporting structures before clinical and radiological signs document a full-blown lesion. MMP-8 is an endopeptidase synthesized in the latent pro-form and activated to its enzymatic form (aMMP-8) in the extracellular space or on the cell surface [62,63]. This enzyme acts on the various types of collagens and the extracellular matrix, degrading their proteins and thus further activating the migration of inflammatory cells and the consequent tissue destruction [64,65]. Several studies have highlighted a direct correlation between the degree of peri-implant tissue inflammatory lesion and the concentration of aMMP-8 in PISF [22,66,67]. Therefore, determining the PISF aMMP-8 concentration has been proposed as a diagnostic tool for identifying the degree of tissue inflammation, particularly in the early inflammatory phase when clinical signs of injury are not yet clearly evident [16,27].

The aMMP-8 cutoff thresholds used by the authors to define risk classes (<20 ng/mL, 20–80 ng/mL, >80 ng/mL) derive from a study by Lähteenmäki et al. [28]. By analyzing the utility of the aMMP-8-PoC/chairside enzyme assay as a real-time quantitative chairside diagnostic tool for peri-implant diagnosis, as well as evaluating potentially developing and ongoing risk compared to traditional clinical methods, the authors found that the greatest agreement between the aMMP-8-PoC quantitative and visual assays was achieved by setting two thresholds: aMMP-8 = 20 and 80 ng/mL. They therefore suggested using intrasulcular aMMP-8 concentrations of <20 ng/mL, ≥20 < 80 ng/mL, and ≥80 ng/mL to define low, moderate, and high-risk thresholds, respectively.

In a previous study, the same patient sample as the present study was used by the authors to validate the BIGR score [26]. The individual parameters hazard ratio (HR) comparison for P events showed a higher value (HR = 6.21, *p* = 0.003) for aMMP-8 > 80 ng/mL, allowed by history of stage II, III and IV periodontitis (HR = 5.19 (*p* = 0.006), while the other parameters individually were not found to be significantly associated with P, thus showing the PISF aMMP-8 concentration to be of better prognostic significance. In the present study, the prognostic value of the BGIR was compared—within the same patient sample—with other indices proposed in the literature, such as the IDRA and PiRA.

Previous studies evaluating the relationship between intrasulcular levels of MMP-8 and peri-implant health status have suggested that the aMMP-8 level should be initially measured at the time of crown delivery (baseline), and the point-of-care test should be repeated at every oral hygiene check-up, along with a reassessment of modifiable risk factors and patient remotivation. This could allow the identification of early and often invisible alterations of the implant support structures before clinical and radiological signs document a complete lesion, and place each patient in the appropriate recall frequency category based on their highest risk score. If worsening of the peri-implant tissue condition is detected during the follow-up period, evidenced by an increase in the aMMP-8 level, the clinician should intervene to reduce or eliminate modifiable risk factors (oral hygiene status, smoking, systemic diseases, prosthetic design related to home-care hygiene procedures and patient dexterity) and remotivate the patient to achieve greater compliance with SPT if this has been deficient. If such interventions are not possible, the patient should be promptly moved to a higher risk category with more stringent intervals before progressive loss of peri-implant support occurs. If the aMMP-8 level changes during the follow-up period, patients should be assigned to another risk category: if the test yields a worse result, the patient should be immediately placed on a more stringent maintenance regime, whereas if it yields a better result, they should be moved to a less stringent maintenance category. If the improvement is slight, the patient should be moved to a less restrictive category only after confirmation of the reduction in the aMMP-8 level at the next visit.

Some limitations are present in the current study, suggesting caution in extrapolating the results:

(1) The retrospective design entails selection bias and possible incomplete or missing data. Furthermore, confounding variables are more difficult to identify, control for, or account for, since both assignment and exposure occurred in the past.
(2) The single-center setting entails limited external validity, as the results reflect the population, protocols, and specific characteristics of only one clinical center and cannot easily be generalized to other settings.
(3) The potential excessive homogeneity and the relatively small sample size represent other limitations, as enrolled patients originate from a single socio-demographic or clinical setting, which could reduce biological variability, limiting generalizability to other contexts.
(4) The use of one implant system.
(5) It is also necessary to underline that the aMMP-8 cutoff thresholds proposed are derived primarily from a single study, while the literature reports other predictive cutoff thresholds for the PISF aMMP-8 concentration to differentiate between healthy and diseased peri-implant tissues. Therefore, specifically orientated multicenter longitudinal prospective studies, involving patients with different dental implants and with different levels of risk at baseline and/or during the follow-up period, are needed to confirm the results of the present study.

## 5. Conclusions

In conclusion, despite the above-mentioned limitations, data collected in the current study showed that patients’ BGIR scores, compared to IDRA and PiRA, during follow-up might represent a better tool to assess which patients are at high risk of developing P.

## Figures and Tables

**Table 1:** Patient data at BIGR calculation time.

BGIR-Related Data			n. (%)
Factors to be considered	aMMP-8 level	<20 ng/mL	58 (72.5%)
		20–80 ng/mL	14 (17.5%)
		>80 ng/mL	8 (10%)
	Oral hygiene status	Good	53 (66.2%)
		Moderate	24 (30.1%)
		High	3 (3.7%)
	History of periodontitis	No	20 (25%)
		Stage II—Grade A/B	13 (16.2%)
		Stage III—Grade A/B	34 (42.5%)
		Stage III—Grade C	6 (7.5%)
		Stage IV	7 (8.8%)
	Smoking	No	39 (48.7%)
		Past smoker	16 (20%)
		Smoker	25 (31.3%)
	Systemic diseases	No	62 (77.6%)
		No/good control	11 (13.7%)
		Yes	7 (8.7%)
	Prosthetic design related to homecare hygiene procedure	EA </= 30° and concave emerging profile Easy access	46 (57.5%)
		EA > 30° regardless of concave or convex profile Limited access	21 (26.2%)
		EA > 30° and convex profile Difficult access	13 (16.3%)
	Patient dexterity assessment	Good	58 (72.5%)
		Limited	12 (15%)
		Compromised	10 (12.5%)
	SCP compliance	Good	62 (77.5%)
		Poor	18 (22.5%)

**Table 2:** Patient data at IDRA calculation time.

IDRA-Related Data			n. (%)
Factors to be considered	% sites with BoP	<10%	10 (12.5%)
		>10 < 25%	51 (63.5%)
		>25%	19 (21.25%)
	PD	≥5 mm	25 (31.2%)
		<5 mm	55 (68.8%)
	History of periodontitis	No	20 (25%)
		Stage II—Grade A/B	13 (16.2%)
		Stage III—Grade A/B	34 (42.5%)
		Stage III—Grade C	6 (7.5%)
		Stage IV	7 (8.8%)
	Smoking	No	39 (48.7%)
		Past smoker	16 (20%)
		Occasional smoker	4 (5%)
		Smoker	21 (26.3%)
	Implant prosthesis-related factors	Well-fitting, cleanable, screw-retained or with no excess cement prosthesis	41 (51.2%)
		Prosthesis with compromised fit but accessible supra-mucosal margins	12 (15%)
		Uncleanable poorly fitting prosthesis, and/or presence of excess submucosal cement	27 (33.8%)
	Periodontal bone loss in relation to the patient’s age	>1	13 (16.2%)
		<1	67 (83.8)
	Distance from RM of the implant-supported prosthesis to the bone:	>1.5 mm	58 (72.5%)
		From 1.5 mm to 2.5 mm	12 (15%)
		<1.5 mm	10 (12.5%)
	SPT	Good	62 (77.5%)
		Poor	18 (22.5%)

**Table 3:** Patient data at PiRA calculation time.

PiRA-Related Data			n. (%)
Factors to be considered	Full mouth bleeding score	Percentage of sites displaying BoP < 9	10 (12.5%)
		Percentage of sites displaying BoP > 9 ≤ 19;	48 (60%)
		Percentage of sites displaying BoP > 20	22 (27.5%)
	PD	≥5 mm	38 (32.5%)
		<5 mm	42 (52.5%)
	History of periodontitis	No	20 (25%)
		Stage II—Grade A/B	13 (16.2%)
		Stage III—Grade A/B	34 (42.5%)
		Stage III—Grade C	6 (7.5%)
		Stage IV	7 (8.8%)
	Smoking	No	39 (48.7%)
		Past smoker	16 (20%)
		<19 cigarettes/day	9 (11.3%)
		>20 cigarettes/day	16 (20%)
	Number of teeth lost	≤4	18 (22.5%)
		>4 ≤ 8	39 (48.7%)
		>8	23 (28.8%)
	Periodontitis susceptibility: (bone loss/age ratio)	>1	13 (16.3%)
		<1	67 (83.8%)
	General health	No	62 (77.6%)
		No/good control	11 (13.7%)
		Yes	7 (8.7%)
	Recall frequency	≥6	46 (57.5%)
		>6 ≤ 8	16 (20%)
		>12	18 (22.5%)

## Data Availability

The data are available from the corresponding author(s) upon reasonable request.

## Abbreviations

The following abbreviations are used in this manuscript:

BGIR	Biomarker-guided implant risk
IDRA	Implant disease risk assessment
PiRA	Peri-implantitis risk assessment
ROC	Receiver operating characteristic
PISF	Peri-implant fluid
aMMP-8	Active metalloproteinase-8
BoP	Bleeding on probing
PD	Probing depth
CAL	Clinical attachment loss
POC	Point-of-Care
